# “Value my culture, value me”: a case for culturally relevant mentoring in medical education and academic medicine

**DOI:** 10.1186/s12909-023-04148-w

**Published:** 2023-04-11

**Authors:** Sylk Sotto-Santiago, Jacqueline Mac, Inginia Genao

**Affiliations:** 1 Department of Medicine, Indiana University School of Medicine, 545 Barnhill Drive, Emerson Hall 305, Indianapolis, IN 46202 USA; 2 Indiana Clinical and Translational Sciences Institute, Indianapolis, USA; 3 Indiana University Simon Comprehensive Cancer Center, Indianapolis, USA; 4 Department of Counseling and Higher Education, Northern Illinois University, Dekalb, IL USA; 5 Pennsylvania State College of Medicine, Hershey, USA

**Keywords:** Mentoring, Cultural relevance, Cultural humility, Historically underrepresented, Diversity

## Abstract

**Introduction:**

Mentoring programs are one mechanism used to increase diversity and participation of historically underrepresented groups in academic medicine. However, more knowledge is needed about the mentoring experiences and how culturally relevant concepts and perspectives may influence diverse students, trainees, and faculty success. This case study utilized the Culturally Engaging Campus Environments (CECE) model which examines the experiences of students in higher education. We used this model to examine the mentoring experiences of Black and Latine faculty and offer practical implications for the medical education continuum.

**Methods:**

Our research approach is best understood through qualitative inquiry stemming from a single-case study which allowed for in-depth understanding of the contexts informing the phenomenon. Phenomenology is well positioned to contribute to understanding science and health professions. Selection criteria included individuals who self-identified as Black or Latine; inclusive of all faculty ranks and tracks. This analysis focuses on 8 semi-structured interviews, averaging 3 h in length.

**Results:**

Findings centered on the area of cultural relevance, and participant narratives revealed the connection of mentoring with cultural familiarity, culturally relevant knowledge, cultural service and engagement, and cultural validation.

**Conclusion:**

The use of cultural relevance indicators can inform the creation and evolution of mentoring programs towards holistic support of historically underrepresented trainees and faculty. Implications also focus on the development of mentors and championing the incorporation of cultural humility in the mentoring process. The implications in praxis offers the possibility for a new framework for culturally relevant mentoring (CRM). Through this framework we aim to enhance and facilitate inclusive learning environments and career development.

## Introduction

Even though faculty diversity in higher education has been increasing, it has not increased at a rate similar to the diversity of learners [1]. For example, the Association for American Medical Colleges (AAMC) showed that faculty at the Assistant Professor ranks had higher proportions of Black/African American (4%) and Hispanic/Latine (2.5%) individuals compared to their White counterparts. The data suggest mobility barriers within promotion and tenure structures. Furthermore, Black and Hispanic/Latine faculty are less likely to be in tenured or tenure-track positions; are concentrated in lower ranks, earn less, and less likely to be promoted and achieve tenure [2]. Early studies also showed that these groups were leaving full-time faculty appointments at a higher rate than men and White faculty members [2].

Mentorship has grown as a tool for everyone in academia and is often considered a critical part of historically underrepresented (HU) groups’ success [3]. However, the mentoring literature does not demonstrate a unified definition, definite “best” practices or conclusive understanding of formal mentoring programs. A plethora of mentoring programs convey a number of endorsements that suggest great progress in mentorship of early career faculty, pre-faculty, and learners. Yet, most programs do not address the numerous barriers disproportionately experienced by historically underrepresented faculty and learners [4]. We posit that culturally relevant mentoring must be in place in order to help break barriers to diversifying the academic medicine workforce. We define academic medicine as a sector of higher education that encompasses a traditional tripartite missions of research, patient-care (traditionally a health system), and education (graduate and/or medical school). For example, scholars have shared that HU faculty have experiences with racism, discrimination, and (micro)aggressions; cultural taxation and tokenism; bias in recruitment, promotion, and tenure; and challenges to their credibility and expertise compared to non-HU faculty [5]. HU learners have difficulty establishing support network and report lower satisfaction with the learning environment. As HU faculty, they are also facing racial discrimination in clinical environments by fellow peers, residents, faculty, and patients. As a result of these experiences, they are less likely to seek assistance [6].

Mentoring literature in support of HU faculty shares several themes: (1) the recruitment of highly qualified and diverse faculty; (2) the retention of high quality and diverse faculty; (3) the benefits to the institution; and (4) a proclaimed alignment with an institution’s mission, culture and norms [7]. Clearly, if these mentoring programs were evaluated based on the success of recruitment, retention and advancement of historically underrepresented groups, we should have seen remarkable increases in the number of Black and Hispanic/Latine faculty in higher education and academic medicine [4, 8].

Due to lower numbers of HU faculty and learners in medicine, it is challenging to build mentoring relationships that connect individuals with similar backgrounds, hence making cross-cultural relationships and culturally relevant mentoring critical. There are increasing calls for evidence-based approaches to training and other interventions to address cultural diversity and to not ignore the realities of racism in the biomedical and health sciences, particularly in mentoring experiences [9]. Current mentoring models are not sufficient for tackling issues such as race, ethnicity, gender, power, and privilege in mentoring relationships. We believe that the majority of mentoring programs have ignored relationships and environments that are responsive to historically underrepresented groups’ needs. For this reason we analyze these experiences through a lens that considers influences and success amongst diverse populations, such as culturally engaging campus environments (CECE, pronounced see-see) [10].

Our goal was to examine the experiences and perspectives of Black and Hispanic/Latine^1^ faculty, specifically focusing this phenomenological analysis on mentorship. The implications in praxis offers the possibility for a new framework for culturally relevant mentoring (CRM). Its foundation is in cultural relevance and responsive mentoring explored through the CECE framework. In doing so, we distilled major themes and learned more about opportunities to enhance and facilitate HU faculty career advancement through cross-cultural mentorship. We begin with a discussion of the conceptual framework.

### Culturally engaging campus environments: a conceptual framework

Culturally engaging campus environments (CECE) are institutional environments that reflect and are responsive to the identities of culturally diverse student populations [10]. Research indicates that these types of college and university environments lead to more positive individual experiences and higher levels of success among diverse learners [10]. Specifically, the Culturally Engaging Campus Environment (CECE; pronounced see-see) suggests that a number of external influences shape individual influences and success among racially diverse learner populations [10]. The CECE model includes nine indicators that characterize these environments: Cultural Familiarity, Culturally Relevant Knowledge, Cultural Community Service, Cultural Validation, Meaningful Cross-Cultural Engagement, Collectivist Cultural Orientations, Humanized Educational Environments, Proactive Philosophies, and Holistic Support.

Cultural familiarity suggests that the extent in which learners have the ability and opportunities to connect with faculty, staff and peers with whom they share commonalities are connected with a higher likelihood of success [10]. Culturally relevant knowledge refers to the ability of higher education institutions to provide opportunities for learners to “cultivate, sustain, and increase knowledge of their cultures and communities of origin,” and by doing so, positively impacting their success [10]. Cultural community service suggests that culturally relevant community service impacts the experiences and success of racially diverse learner populations [10]. Meaningful cross-cultural engagement offers and promotes many positive outcomes in college while Collectivist Cultural Orientations create spaces that counter individualist orientations. Culturally validating environments positively affect success by affirming the learners’ cultural backgrounds and identities [10]. Humanized educational environments refers to environments created by caring and committed institutional agents. Lastly, proactive philosophies and holistic support provide positive vision of success among racially diverse learners, and the resources and support necessary for success respectively [10].

The intent of the CECE model is to provide indicators that may guide institutional action in a positive direction while stimulating discourse and a potential guiding framework for committed institutions to be explicit about the success of racially diverse populations. With this purpose, CECE indicators offer an opportunity to study the application of these elements into the mentoring experiences of HU faculty, therefore also impacting learners.

### Study purpose and research questions

In this article, we seek to examine the mentoring experiences of Black and Hispanic/Latine faculty in academic medicine. Data in this article comes from a larger study about the experiences of Black and Hispanic/Latine faculty primarily examining socialization, mentoring, and faculty development in academic medicine [4]. Two overarching research questions guide this article and analysis: What are the mentorship experiences of Black and Hispanic/Latine faculty in academic medicine? How may these experiences inform mentoring models that are culturally relevant?

### Methodology

Our research approach is best understood through qualitative inquiry stemming from a single-case study which allowed for in-depth understanding of the contexts informing the phenomenon of interest [4, 8, 11]. This analysis focuses on phenomenology, which is very well positioned to contribute to the health professions and academic medicine [12]. For example, in this phenomenological analysis we attempt to understand the lived experiences of individuals experiencing a particular phenom and through the voices of Black and Hispanic/Latine faculty we offer a shared understanding among the participants [13]. The study was approved as an expedited protocol ( #1510364481) by Indiana University IRB.

### Case context

This research was conducted at Midwest School of Medicine (MSOM) in the Midwest region of the United States. MSOM is currently one of the largest schools of medicine in the country and considered a national leader in medical research and education. A core diversity emphasis of the institution is to advance Black and Hispanic/Latine faculty through promotion and leadership positions. At the time this study was conducted, Black and Hispanic/Latine faculty represent an estimated < 4% each, of its full-time faculty (MSOM).

### Participant selection

Purposeful and criterion sampling of participants helped identify those with firsthand knowledge and experience [14]. Criteria included individuals who self-identified as Black or Hispanic/Latine; inclusive of all faculty ranks and tracks. The call for participants was circulated widely across the institution.

The final sample of eight faculty consisted of tenured and non-tenured Black and Latino faculty who held the rank of Assistant, Associate, and/or Full Professor. The U.S. higher education system has several forms of faculty appointments including adjunct and visiting appointments which denote a part-time or temporary status. Others such as lecture and instructor appointments depend on the institution and may indicate teaching responsibilities. Traditionally, Assistant Professor may be considered an entry point to the professorate and depending on the institution it may indicate multiple responsibilities across missions. In academic medicine, this may indicate teaching, research, and clinical or administrative service responsibilities. This classification has promotion opportunities to Associate and Full Professor.

Each participant has been given the following pseudonyms: Rosana, Jessika, Henok, Alberto, Francisco, Evelio, Blair, and Elizabeth. Table 1 summarizes participant characteristics. Due to the confidentiality promised to participants, providing additional demographic details about each participant may risk identification [2].

**Table 1:** Participants

Pseudonym	Faculty Rank	Identity (Self-Identification)
		*Gender: Woman*
Blair	Associate Professor	Latina/Mexican–American
Elizabeth	Assistant Professor	Black
Rosana	Assistant Professor	Black/Multiracial
Jessika	Assistant Professor	Black
		*Gender: Man*
Henok	Associate Professor	Latino/ Multiracial
Francisco	Professor	Latino/Naturalized US Citizen
Evelio	Professor	Black
Alberto	Assistant Professor	Latino/Naturalized US Citizen

### Data collection and analysis

This analysis focuses on 8 semi-structured interviews, ranging from 2.5–3 h in length. All interviews were recorded and professionally transcribed as part of a previous study focusing on Black and Hispanic/Latine experiences in academic medicine exploring the socialization, mentoring, and faculty development experiences [4]. For this article, we chose a phenomenological analysis approach. Phenomenology can be defined as an approach to research that seeks to describe the essence of a phenomenon by exploring it from the perspective of those who have experienced it both in terms of what was experienced and how it was experienced [12, 15].

**Fig. 1:**
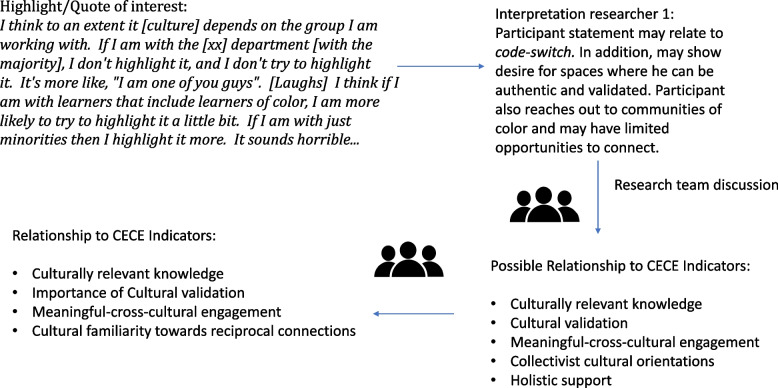
Example of phenomenological reduction process

The first step in this analysis was the phenomenological reduction process [16]. In order to carry out this step the researcher needs to be receptive to every statement and as part of the epoché^2^ process, statements that referred to the CECE indicators were extracted off the transcript. Meaning units were gathered together to form core themes. These themes were organized based on commonality of interpretation underlying the experiences. Figure 1 briefly demonstrates this process.

These initial themes included: mentoring roles, institutional navigator, positive mentoring, negative mentoring, informal mentoring, identity as mentors, relationship building, validation and affirmation, service, equity and inclusion, and cultural competence. We have included participant narratives to convey their unique perceptions of the phenomenon investigated. The composite textural descriptions enabled each participant to be represented under specific themes and indicators.

### Researcher positionality

To choose a phenomenological approach requires the scholar to reflect on their own positionality. As we also consider ourselves members of historically underrepresented groups in academia, we have always been inspired by HU faculty presence and stories. They are the most influential reasons to perform this scholarship and to engage in similar career paths. The number of underrepresented groups in academic medicine is especially dismal, yet we are constantly struggling to impact the very pipeline that represents the majority healthcare workforce that focuses on caring for underserved, underresourced and vulnerable populations.

### Trustworthiness

Lincoln and Guba suggested that trustworthiness of a qualitative research study is critical in evaluating its merits [17]. Trustworthiness involves establishing: credibility, defined as confidence in the findings; transferability, demonstrating some applicability; dependability which shows consistency; and confirmability. Participants were offered the opportunity to review interview summaries. This provided participants with the ability to review and revise their own statements. This step aligned with confirmability and the practice of participant checking, which is the practice of seeking feedback and checking the accuracy of the researcher’s interpretations [18]. In validating, a researcher must question their own findings and integrate validation in every stage of the construction of knowledge [19].

### Findings

In this section, we share themes that illuminate the relationship between culturally engaging environments and mentoring. Cultural relevance was the unifying theme that represented a lack of attention to and difficulty not only in relating these experiences, but how participants made sense of the experience itself. The CECE framework provided an opportunity to consider the relationship between culturally relevant environments and mentoring themes. For example, participants’ responses regarding the areas of cultural familiarity, cultural service and culturally relevant knowledge offered a glance that culturally relevant environments are important to Black and Hispanic/Latine faculty career paths in higher education and academic medicine.

### Cultural familiarity-lack of opportunity to connect with others

Participants in this study found it challenging to find colleagues and others on campus with similar backgrounds hence limiting the exposure and opportunities to interact with individuals like themselves. They recognized that this was an issue based on the number of other HU faculty at the institution, but also expressed the importance that this familiarity has on their satisfaction, sense of belonging, retention, leadership opportunities, and so forth. In addition, participants lacked a space in which they could connect with people from their respective communities. This can be seen in Rosana’s reflection:

So none on campus so much, I think I was fortunate because in my own department, there were a few people (of color). So we had a fair number in our department but on the campus it is not easy. I think it's really difficult to find other people of color, especially when you start putting events together to try to network with people and try to expose (medical) students to people, it's really difficult to identify and to find out who’s who, it's difficult on campus, and everything is very siloed here.

Further, this lack of culturally familiarity poses difficulty in identifying and establishing possible mentoring relationships in which individuals feel reciprocal connections. The search for mentors that understand the experiences and challenges but above all can help guide and navigate the political environment of the institution alone might be a sign of resilience and persistence. It is important to note that cultural familiarity does not need to only be attained by sharing a cultural background, it may include commonalities that can be shared amongst groups.

### Culturally relevant knowledge-limited opportunities to access and expression

Participants expressed there were not enough opportunities to learn or highlight their own cultures or share such knowledge. As a result, participants expressed hesitancy to sharing their cultures in relationships. One participant, Henok, in particular spoke about a “spectrum”:

I think to an extent it [culture] depends on the group I am working with. If I am with the [xx] department [with the majority], I don't highlight it, and I don't try to highlight it. It's more like, "I am one of you guys". [Laughs] I think if I am with learners that include learners of color, I am more likely to try to highlight it a little bit. If I am with just minorities then I highlight it more. It sounds horrible...

Even though sharing one’s culture is an important component of the mentoring relationship, some HU faculty may not feel comfortable highlighting their culture in efforts to appear assimilated or fear of rejection. Institutions do not facilitate environments in which culturally relevant knowledge can be shared with the majority. Specifically, as with learners, this space would increase the likelihood of success and stronger connection to their institution. As it stands, Henok is only able to share his culture with other HU faculty; otherwise, Henok may model his behavior as that of the majority, sustaining that “assimilation” is necessary and practically required.

### Commitment to cultural community service not leveraged

Participants often spoke about service, specifically service to their cultural communities or to give back in ways that improve the lives of those in their communities. It was clear that participants valued service in ways that gave them personal satisfaction. However, participants simultaneously experienced tension with the notion that this service should be tied to an academic currency. Otherwise, their success and career advancement as Black and Hispanic/Latine faculty can be further suppressed. Service and social justice are present values for all participants in the way they talk about what motivated them to a career in science, academic medicine, to work with patients, to teach and mentor learners. For example, Jessika talked about how social justice is at the center of her work.

What might motivate me is about social justice and issues around sort of health and disparity and the impact of race and class and culture on health and race… It's all of that stuff. It's not apparent, but it's at the center of what I do… And so, when I sort of step back in terms of like big picture, like what I want to be accomplishing in the world, I wanted to be like... A safer place for Black women and girls, not just exclusively that, but that sort of I want to be doing work that is actually having impact. And I think that that's become the question mark with regard to trajectory, is whether or not the incremental change and impact that you can have in academic settings is one that is going to kind of afford me the ability to do that work in the most meaningful or effective way.

These statements support Black and Hispanic/Latine values that are often not explored in mentoring relationships or rewarded by the institution through the promotion and tenure process. In addition, it demonstrates a forced departure from collectivist orientations, as Henok shares:

If you are interested in service, how can you utilize that service and make that service in a way that it can actually get you promoted. It's something that I sometimes still, or faculty of color struggle with.….

Participants do not feel rewarded or incentivized by higher education and academic medicine and more specifically, the institution when it came to mentoring. There appears to be a disconnect between the meaning of service for Black and Hispanic/Latine faculty and the institution. Jessika discusses these complexities by sharing that she struggled with feelings of selfishness in evaluating her service.

You get these messages about how you have to be selfish with your time early on and that you're not going to be any good to all these people that you want to help or if you don't get promoted and you have to leave [laughs]. And so, I mean those are very explicit messages...…I think if they are ways to actually reward in a real way around promotion and tenure doing that work of advising and mentoring, doing that work of community service and community engagement. If those things were things that could be counted toward promotion it would just create better alignment but a lot of that feels like free service that you're giving away that isn't going to ultimately translate or be accounted for in the equation when they're looking at the number of publications the real productivity measures.

### Cultural validation

The question of validation was always answered with some trepidation. Overall, participants had mixed responses about feeling validated through their cultural backgrounds, knowledge, and identities of diverse students. The answers were carefully crafted. The perspective was that many of them ultimately felt neither validated nor valued by the school and the institution. As Francisco stated “if you value my culture, you value me**”**. CECE’s culturally validating environments positively affect success by validating the learners’ cultural backgrounds and identities [10]. For example, scholars have documented how culturally validating environments can increase engagement, resilience, and sense of belonging, which ultimately contribute to motivation [20]. HU faculty can also validate minoritized learners’ cultural backgrounds and experiences and in that manner decrease the cultural conflict experienced by those learners of color [21]. Same could hold true for HU faculty. Alberto offered a good summary of this notion of value at the institution:

If somehow, we identify who underrepresented faculty are, what are their potential, interests and how can they help the institution, maybe can do better, maybe they will feel valued, not only because of how good they are in what they do, but how good they are because of who they are.

By giving Black and Hispanic/Latine faculty a similar opportunity to acknowledge and validate their own cultural background and validate the strengths they provide to the institutions we may also decrease their own cultural dissonance. Enhancing cross-cultural mentoring can contribute towards these efforts.

## Discussion

While mentoring is an area that has been extensively studied and at times programmatically exhausted, we still do not have an ideal model supporting Black and Hispanic/Latine faculty. Certainly, if mentoring programs were indeed as successful as professed, we would surely have seen a remarkable increase in the number of Black, Hispanic/Latine and other underrepresented groups in academia. Yet, that is not the case, and this analysis aimed to understand the experiences of Black and Hispanic/Latine faculty through the mentoring received and provided. By examining lived experiences, new meanings and appreciations can be developed to inform and re-orient how we understand that experience [22–24].

As programs also focused on developing mentors, they need to also acknowledge that at this point, being mentored by someone from similar background, might be at odds with what the diversity-related numbers indicate in academia. It is also a mistake to continue to support arguments such as “if only there were more senior faculty of color”. Yes, we need more HU faculty at senior levels and leadership, but this is not the most salient reason for low numbers in academic medicine; it is about the work and learning environments. In the interest of effectively using mentorship as ways to recruit, retain, promote career advancement and development, the themes in this analysis highlighted the need for more than career advice and mentoring relationships that understand the HU faculty assets, strength, knowledge and potential; mentorship identity is grounded by value systems committed to service; and realizing Black and Hispanic/Latine faculty will be most likely mentored by individuals of different backgrounds. Because of these elements, we need to reenvision mentoring; culturally relevant mentoring is necessary [25].

While the CECE framework does not specifically address mentoring, there are indicators that clearly relate and would enhance cultural relevance in mentoring. Black and Hispanic/Latine faculty did not state that individuals under the mentorship umbrella came from similar backgrounds, yet a collection of mentors provided what they need in order to be this successful. Therefore, Black and Hispanic/Latine faculty suggest that cross-cultural mentoring is happening in ways that positively impact their career. Institutional leaders and faculty and professional developers’ efforts around mentorship should also focus greatly on the development of mentors and protégées.

We highlight two major implications for practice. First, the use of culturally relevant indicators, such as those highlighted under the CECE model: cultural familiarity, culturally relevant knowledge, cultural engagement and service, and cultural validation, can inform the creation and evolution of mentoring programs. Mentorship is essential and these indicators reemphasize that there are a set of skills that mentors can learn, practice, and continuously enhance. Widely used programs such as those by the National Research Mentoring Network (NRMN) have various resources including unconscious bias and antiracist resources and focus on the development of mentoring skills. Programs like these can be enhanced by incorporating modules, examples, and intentional discussions that specifically speak to the importance of HU faculty sharing cultural knowledge, collaboratively engaging in service, and how validation and value increase sense of belonging in academia.

Our second implication focuses on the development of mentors. Black and Hispanic/Latine faculty demonstrated the importance of establishing mentoring relationships in which individuals feel reciprocal cultural connections. Mentors to HU faculty and learners can practice cultural familiarity in the way they develop relationships, in the ability to connect with individuals and identify commonalities. Cultural familiarity extends to the way mentors socialize faculty into the academy, in introducing them to the community, in serving and introducing them to a mentoring network of champions, sponsors, role models, advisors, institutional agents, navigators, etc. Cultural familiarity for cross-cultural mentorship shows in the development of allyship, upstanders, and accomplices in advancing racial equity. Culturally relevant knowledge practices would encourage the ability to share experiences that help increase mutual knowledge, educating mentors and protégés on topics important to inclusion. For example, development would expand to implicit or unconscious bias, cultural taxation, importance of service and its burden, microaggression, gender disparities, spirituality, LGBTQ + issues, anti-racist pedagogies and practices, and cultural humility. Cultural community service would value every type of good mentorship provided to learners and peers. It would recognize service that leads to promotion and tenure, service that promotes social justice and equity, civic engagement, etc. Other forms of cross-cultural engagement by mentors and protégés would be represented in opportunities to demonstrate true interest and authenticity via participation and engagement is cultural activities.

Proactive philosophies and holistic support provide a positive vision of success among racially diverse learners and the resources necessary for success [10]. Hence in terms of mentoring, this would be represented in the development of individualized career plans, mentoring plan approaches, informal mentoring and building mentoring networks; in addition to institutional resources that recognize the importance of protected time and faculty development programs.

The CECE model, although enriched with culturally relevant elements could be further enriched by, perhaps, one of the most fundamental values in health care professions, the concept of cultural humility. Cultural humility as described by Tervalon and Murray-Garcia is a commitment to actively engage in a life-long process of humility, continually engaged in self-reflection and self-critique [26]. Specific to cultural humility training in medicine, it demands to check the power imbalances that exist by practicing patient-focused care. What if we applied cultural humility in mentoring in ways that directly put the mentee or protégée as the focus of our relationship? Cultural humility offers the opportunity to reflect on our mentoring experiences in order to improve the experiences that we provide to others. Being inclusive and humble, means being respectful, curious and engaging in a phenomenal mentoring relationship.

The incorporation of CECE indicators and cultural humility would also facilitate application to global environments. Mentoring is one important component in a larger strategy to build inclusive, cohesive, and collaborative higher education communities, and create a continuously engaging culture that can adapt to global change. However, we have been mentoring towards an “outcome” in academia from helping individuals to achieve research funding to encouraging innovative teaching, less effort has been devoted to assist HU faculty in navigating the socio-political environments of the institution, and most importantly, applying a holistic approach to mentoring.

The main limitation of this study is that the interpretation are bounded by the context of the case study, situation, subjectivity of both the participants and researchers. Although it provides the ability to better explore the application of CECE; its results does not represent a longitudinal view of mentorship. However, the strengths of this study outweigh this. First, it expands the current higher education literature to include academic medicine. Second, it is the first study to apply CECE in academic medicine. Third, it begins to highlight the importance of cultural relevance, at par with cultural humility, cultural competence, responsive and socially responsible STEM, medical education and environment.

## Conclusion

Ideally, the development of mentors that support a diverse population would emphasize the importance of embracing culturally relevance indicators. Items like Cultural Familiarity, Cultural Knowledge, Cultural Community Service, Cultural Validation, Meaningful Cross-Cultural Engagement, Collectivist Cultural Orientations, Humanized Educational Environments, Proactive Philosophies, and Holistic Support should not be exclusive from one another. It is important to highlight that the ability and opportunities to connect with HU faculty and learners can only be elevated by the opportunity to share themselves as they are. This of course is important throughout the mentor-protégée relationship. It should be as important for both parties to share such knowledge, in ways that enrich both sides of this relationship.

Cultural humility also serves as a form of validation in ways that demonstrate true interest and authenticity via participation and engagement. As one participating stated: “Value my culture, value me.” Validation of individual’s accomplishments and backgrounds is not a matter of attention or recognition. It encourages self-valuation and confidence, but it also builds that sense of belonging, an institutional message of inclusion, of value in faculty development and medical education alike.

## Data Availability

The data sets used/analyzed during the study are available from the corresponding author on reasonable request.

## Footnotes

1 While Latino is a masculine identifier in the Spanish language, for the purposes of this article, we will use the term(s). Latine as a gender-inclusive word that is inclusive of Latinas, Latinos, and those with non-binary, non-conforming, and gender fluid gender identities. Scholars may also use the term Latinx as an inclusive term for members of these groups.

2 Epoché, allows for empathy and connection, not elimination, replacement or substitution of perceived researcher bias. In connection with bracketing in phenomenological research, it is described as a process involved in the researcher’s awareness of assumptions in order to explain a phenomenon in terms of its own meaning.
